# Supplementary material for: Radiogenomics of C9orf72 Expansion Carriers Reveals Global Transposable Element Derepression and Enables Prediction of Thalamic Atrophy and Clinical Impairment
Source: J Neurosci. 2023 Jan 11;43(2):333–45. doi: 10.1523/JNEUROSCI.1448-22.2022 (PMC9838702; doi:10.1523/JNEUROSCI.1448-22.2022)
Supplement: Figure 1-1 — Thalamic volume differences in C9orf72 HRE carriers compared to controls covarying for total intracranial volume. Comparisons of thalamic nuclei volumes in C9orf72 HRE carriers versus controls. Results from all 50 thalamic nuclei volumes estimated using FreeSurfer 7.1 software are shown above with p values shown before and after FDR correction for multiple testing. All regression analysis covaried for clinical severity (as estimated by CDR-SB score), age, sex, education, MRI scanner type (1.5T, 3T, or 4T), and total intracranial volume. L, Left. Download Figure 1-1, DOCX file. [file ns-JN-RM-1448-22-s01.docx]

Figure 1-1: Thalamic volume differences in *C9orf72* HRE carriers compared to controls covarying for total intracranial volume

| Region | Beta | Standard Error | *P*-value | FDR *P*-Value |
| --- | --- | --- | --- | --- |
| R. Mediodorsal Lateral Parvocellular | -56.18 | 8.49 | 6.83E-09 | 3.42E-07 |
| R. Pulvinar Anterior | -38.21 | 6.78 | 3.61E-07 | 6.98E-06 |
| L. Ventral Anterior | -63.61 | 11.36 | 4.19E-07 | 6.98E-06 |
| R. Ventral Anterior | -58.40 | 11.91 | 6.12E-06 | 7.65E-05 |
| L. Lateral Posterior | -31.19 | 6.56 | 1.07E-05 | 1.07E-04 |
| L. Mediodorsal Lateral Parvocellular | -43.04 | 9.29 | 1.67E-05 | 1.31E-04 |
| R. Anteroventral | -33.59 | 7.29 | 1.83E-05 | 1.31E-04 |
| R. Intralaminar Central Medial | -13.59 | 2.99 | 2.33E-05 | 1.46E-04 |
| R. Ventral Anterior Magnocellular | -4.44 | 0.99 | 2.73E-05 | 1.52E-04 |
| L. Ventral Anterior Magnocellular | -4.26 | 0.96 | 3.41E-05 | 1.62E-04 |
| L. Intralaminar Central Medial | -13.52 | 3.05 | 3.56E-05 | 1.62E-04 |
| R. Ventral Lateral Anterior | -67.10 | 15.49 | 4.98E-05 | 1.92E-04 |
| L. Ventral Lateral Anterior | -69.35 | 16.01 | 4.99E-05 | 1.92E-04 |
| L. Anteroventral | -31.08 | 7.27 | 6.11E-05 | 2.18E-04 |
| R. Lateral Posterior | -27.71 | 6.55 | 7.23E-05 | 2.41E-04 |
| L. Laterodorsal | -10.53 | 2.73 | 2.52E-04 | 7.88E-04 |
| R. Pulvinar Medial | -144.58 | 37.70 | 2.77E-04 | 8.15E-04 |
| R. Pulvinar Inferior | -37.64 | 10.31 | 5.08E-04 | 1.34E-03 |
| R. Pulvinar Lateral | -33.73 | 9.24 | 5.08E-04 | 1.34E-03 |
| R. Mediodorsal Medial Magnocellular | -83.10 | 23.65 | 7.91E-04 | 1.98E-03 |
| L. Paracentral | -0.50 | 0.14 | 8.53E-04 | 2.03E-03 |
| L. Pulvinar Anterior | -23.45 | 6.83 | 1.02E-03 | 2.32E-03 |
| R. Ventral Lateral Posterior | -61.59 | 19.99 | 2.97E-03 | 6.46E-03 |
| R. Parafascicular | -6.55 | 2.15 | 3.28E-03 | 6.83E-03 |
| L. Medial Ventral (Reuniens) | -2.28 | 0.78 | 4.44E-03 | 8.88E-03 |
| R. Medial Ventral (Reuniens) | -2.63 | 0.91 | 5.06E-03 | 9.73E-03 |
| L. Pulvinar Inferior | -24.42 | 8.57 | 5.77E-03 | 0.01 |
| R. Lateral Geniculate | -24.84 | 9.01 | 7.47E-03 | 0.01 |
| L. Ventral Lateral Posterior | -57.51 | 21.21 | 8.46E-03 | 0.01 |
| R. Paracentral | -0.40 | 0.15 | 9.94E-03 | 0.02 |
| R. Ventromedial | -3.44 | 1.32 | 0.01 | 0.02 |
| L. Intralaminar Central Lateral | -6.16 | 2.40 | 0.01 | 0.02 |
| R. Laterodorsal | -8.09 | 3.21 | 0.01 | 0.02 |
| L. Pulvinar Medial | -81.61 | 34.24 | 0.02 | 0.03 |
| R. Intralaminar Central Lateral | -5.12 | 2.77 | 0.07 | 0.10 |
| L. Mediodorsal Medial Magnocellular | -50.21 | 28.64 | 0.08 | 0.11 |
| R. Ventral Posterolateral | -54.45 | 31.09 | 0.08 | 0.11 |
| L. Pulvinar Lateral | -16.61 | 9.62 | 0.09 | 0.12 |
| L. Suprageniculate | -3.49 | 2.11 | 0.10 | 0.13 |
| R. Suprageniculate | -2.76 | 1.86 | 0.14 | 0.17 |
| L. Lateral Geniculate | -13.54 | 9.21 | 0.15 | 0.17 |
| L. Parafascicular | -2.77 | 1.92 | 0.15 | 0.17 |
| R. Paratenial | -0.40 | 0.28 | 0.15 | 0.17 |
| R. Intralaminar Centromedian | -11.50 | 8.14 | 0.16 | 0.18 |
| L. Paratenial | 0.25 | 0.29 | 0.39 | 0.43 |
| L. Ventromedial | -0.87 | 1.16 | 0.45 | 0.49 |
| R. Medial Geniculate | -3.86 | 6.84 | 0.57 | 0.61 |
| L. Intralaminar Centromedian | -2.36 | 7.96 | 0.77 | 0.79 |
| L. Medial Geniculate | -1.55 | 5.32 | 0.77 | 0.79 |
| L. Ventral Posterolateral | -0.58 | 28.49 | 0.98 | 0.98 |

Comparisons of thalamic nuclei volumes in *C9orf72* HRE carriers vs. controls. Results from all 50 thalamic nuclei volumes estimated using Freesurfer 7.1 are shown above with *p*-values shown before and after FDR correction for multiple testing. All regression analysis covaried for clinical severity (as estimated by CDR-SB score), age, sex, education, MRI scanner type (1.5T, 3T, or 4T), and total intracranial volume. R. – Right, L. – Left.
