# Supplementary material for: Radiogenomics of C9orf72 Expansion Carriers Reveals Global Transposable Element Derepression and Enables Prediction of Thalamic Atrophy and Clinical Impairment
Source: J Neurosci. 2023 Jan 11;43(2):333–45. doi: 10.1523/JNEUROSCI.1448-22.2022 (PMC9838702; doi:10.1523/JNEUROSCI.1448-22.2022)
Supplement: Figure 2-1 — Thalamic volume differences in C9orf72 HRE carriers compared to controls covarying for total thalamic volume. Sensitivity analyses comparing of thalamic nuclei volumes in C9orf72 HRE carriers versus controls after covarying for total thalamic volume rather than total intracranial volume. Results from all 50 thalamic nuclei volumes estimated using FreeSurfer 7.1 software are shown above with p values shown before and after FDR correction for multiple testing. All regression analysis covaried for clinical severity (as estimated by CDR-SB score), age, sex, education, MRI scanner type (1.5T, 3T, or 4T), and total thalamic volumes. L, Left. Download Figure 2-1, DOCX file. [file ns-JN-RM-1448-22-s02.docx]

Figure 2-1: Thalamic volume differences in *C9orf72* HRE carriers compared to controls covarying for total thalamic volume

| Region | Beta | Standard Error | *P*-Value | FDR *P*-Value |
| --- | --- | --- | --- | --- |
| R. Mediodorsal Lateral Parvocellular | -39.72 | 8.51 | 1.48E-05 | 7.40E-04 |
| L. Paratenial | 1.12 | 0.28 | 1.83E-04 | 4.57E-03 |
| L. Ventral Posterolateral | 95.99 | 26.44 | 5.44E-04 | 9.06E-03 |
| L. Intralaminar Centromedian | 24.68 | 6.98 | 7.31E-04 | 9.14E-03 |
| R. Pulvinar Anterior | -18.30 | 5.72 | 2.08E-03 | 0.02 |
| R. Intralaminar Centromedian | 17.44 | 6.42 | 8.39E-03 | 0.07 |
| L. Ventral Anterior | -26.68 | 10.16 | 0.01 | 0.07 |
| R. Intralaminar Central Medial | -7.68 | 2.94 | 0.01 | 0.07 |
| L. Lateral Posterior | -16.26 | 6.46 | 0.01 | 0.07 |
| R. Anteroventral | -18.40 | 7.40 | 0.02 | 0.07 |
| L. Mediodorsal Lateral Parvocellular | -22.44 | 9.17 | 0.02 | 0.07 |
| L. Ventromedial | 2.64 | 1.09 | 0.02 | 0.07 |
| L. Intralaminar Central Medial | -7.26 | 3.04 | 0.02 | 0.08 |
| R. Ventral Posterolateral | 59.27 | 25.47 | 0.02 | 0.08 |
| L. Anteroventral | -16.13 | 7.29 | 0.03 | 0.10 |
| R. Ventral Anterior | -22.04 | 10.77 | 0.04 | 0.13 |
| L. Laterodorsal | -5.60 | 2.74 | 0.04 | 0.13 |
| R. Ventral Lateral Posterior | 27.00 | 13.44 | 0.05 | 0.13 |
| R. Pulvinar Lateral | -18.06 | 9.78 | 0.07 | 0.18 |
| L. Medial Geniculate | 9.35 | 5.37 | 0.09 | 0.20 |
| R. Lateral Posterior | -11.07 | 6.38 | 0.09 | 0.20 |
| L. Parafascicular | 3.14 | 1.82 | 0.09 | 0.20 |
| R. Paratenial | 0.42 | 0.25 | 0.10 | 0.21 |
| R. Ventral Anterior Magnocellular | -1.16 | 0.75 | 0.13 | 0.26 |
| R. Medial Ventral (Reuniens) | -1.43 | 0.95 | 0.14 | 0.27 |
| R. Medial Geniculate | 9.68 | 6.80 | 0.16 | 0.31 |
| L. Medial Ventral (Reuniens) | -1.11 | 0.80 | 0.17 | 0.31 |
| L. Pulvinar Medial | 38.05 | 27.97 | 0.18 | 0.32 |
| R. Pulvinar Inferior | -13.85 | 10.48 | 0.19 | 0.33 |
| L. Ventral Anterior Magnocellular | -1.01 | 0.79 | 0.20 | 0.34 |
| R. Mediodorsal Medial Magnocellular | -26.96 | 22.47 | 0.23 | 0.38 |
| L. Ventral Lateral Posterior | 19.03 | 16.91 | 0.26 | 0.41 |
| L. Ventral Lateral Anterior | -13.75 | 13.17 | 0.30 | 0.44 |
| L. Lateral Geniculate | 10.11 | 9.74 | 0.30 | 0.44 |
| R. Laterodorsal | -3.46 | 3.36 | 0.31 | 0.44 |
| L. Paracentral | -0.13 | 0.14 | 0.36 | 0.50 |
| L. Pulvinar Anterior | -5.59 | 6.21 | 0.37 | 0.50 |
| R. Pulvinar Medial | -23.80 | 32.71 | 0.47 | 0.62 |
| R. Ventromedial | 0.70 | 1.14 | 0.54 | 0.69 |
| L. Mediodorsal Medial Magnocellular | 13.89 | 27.99 | 0.62 | 0.78 |
| R. Paracentral | 0.05 | 0.13 | 0.69 | 0.84 |
| R. Ventral Lateral Anterior | -4.12 | 11.68 | 0.73 | 0.86 |
| L. Pulvinar Lateral | -3.09 | 10.20 | 0.76 | 0.86 |
| R. Suprageniculate | 0.56 | 1.90 | 0.77 | 0.86 |
| R. Intralaminar Central Lateral | 0.74 | 2.82 | 0.79 | 0.86 |
| L. Pulvinar Inferior | -2.31 | 8.77 | 0.79 | 0.86 |
| R. Parafascicular | -0.34 | 1.84 | 0.85 | 0.88 |
| L. Intralaminar Central Lateral | -0.41 | 2.38 | 0.86 | 0.88 |
| L. Suprageniculate | -0.33 | 2.20 | 0.88 | 0.88 |
| R. Lateral Geniculate | -1.38 | 9.45 | 0.88 | 0.88 |

Sensitivity analyses comparing of thalamic nuclei volumes in *C9orf72* HRE carriers vs. controls after covarying for total thalamic volume rather than total intracranial volume. Results from all 50 thalamic nuclei volumes estimated using Freesurfer 7.1 are shown above with *p*-values shown before and after FDR correction for multiple testing. All regression analysis covaried for clinical severity (as estimated by CDR-SB score), age, sex, education, MRI scanner type (1.5T, 3T, or 4T), and total thalamic volumes. R. – Right, L. – Left.
