# Supplementary material for: Radiogenomics of C9orf72 Expansion Carriers Reveals Global Transposable Element Derepression and Enables Prediction of Thalamic Atrophy and Clinical Impairment
Source: J Neurosci. 2023 Jan 11;43(2):333–45. doi: 10.1523/JNEUROSCI.1448-22.2022 (PMC9838702; doi:10.1523/JNEUROSCI.1448-22.2022)
Supplement: Figure 3-1 — C9orf72 expression in C9orf72 HRE carriers versus controls. Multiple regression analyses demonstrate that C9orf72 expression in C9orf72 HRE carriers versus controls is significantly decreased after covarying for the effects of sex, age, education, clinical severity (as estimated by CDR-SB score), and RNA-seq batch. Download Figure 3-1, DOCX file. [file ns-JN-RM-1448-22-s03.docx]

Figure 3-1: *C9orf72* expression in *C9orf72* HRE carriers vs. controls

| Variable | Beta | Standard Error | *P*-Value |
| --- | --- | --- | --- |
| Sex (male) | 0.07 | 0.21 | 0.72 |
| Age (years) | -0.01 | 0.01 | 0.56 |
| Education (years) | -0.05 | 0.04 | 0.30 |
| CDR-SB score | -0.03 | 0.04 | 0.43 |
| Batch | -0.13 | 0.21 | 0.55 |
| *C9orf72* HRE status (carrier) | -1.09 | 0.30 | 5.56E-04 |

Multiple regression analyses demonstrate that *C9orf72* expression in *C9orf72* HRE carriers vs. controls is significantly decreased after covarying for the effects of sex, age, education, clinical severity (as estimated by CDR-SB score), and RNA-seq batch.
