# Supplementary material for: Radiogenomics of C9orf72 Expansion Carriers Reveals Global Transposable Element Derepression and Enables Prediction of Thalamic Atrophy and Clinical Impairment
Source: J Neurosci. 2023 Jan 11;43(2):333–45. doi: 10.1523/JNEUROSCI.1448-22.2022 (PMC9838702; doi:10.1523/JNEUROSCI.1448-22.2022)
Supplement: Figure 3-2 — Right mediodorsal lateral parvocellular nucleus volumes associate with cortical thicknesses. Associations between right mediodorsal lateral parvocellular nucleus volumes and cortical thicknesses are shown. Results for all 68 cortical regions of interest from the Desikan–Killiany atlas with p values shown before and after FDR correction for multiple testing. All regression analysis covaried for clinical severity (as estimated by CDR-SB score), age, sex, education, MRI scanner type (1.5T, 3T, or 4T), and total intracranial volume. L, Left. Download Figure 3-2, DOCX file. [file ns-JN-RM-1448-22-s04.docx]

Figure 3-2: Right mediodorsal lateral parvocellular nucleus volumes associate with cortical thicknesses

| Region | Beta | Standard Error | *P*-Value | FDR *P*-Value |
| --- | --- | --- | --- | --- |
| L. Pars Triangularis | 1.81E-03 | 4.99E-04 | 5.50E-04 | 0.03 |
| L. Rostral Middle Frontal | 1.70E-03 | 5.07E-04 | 1.30E-03 | 0.03 |
| L. Pars Orbitalis | 2.32E-03 | 7.00E-04 | 1.44E-03 | 0.03 |
| L. Pars Opercularis | 1.65E-03 | 5.30E-04 | 2.73E-03 | 0.05 |
| R. Superior Temporal | 1.31E-03 | 4.71E-04 | 6.79E-03 | 0.08 |
| R. Lingual | 1.14E-03 | 4.12E-04 | 7.06E-03 | 0.08 |
| R. Superior Frontal | 1.31E-03 | 4.79E-04 | 8.07E-03 | 0.08 |
| L. Insula | 1.53E-03 | 5.71E-04 | 9.25E-03 | 0.08 |
| R. Pars Opercularis | 1.28E-03 | 5.03E-04 | 0.01 | 0.08 |
| L. Lateral Orbitofrontal | 1.15E-03 | 4.56E-04 | 0.01 | 0.08 |
| R. Medial Orbitofrontal | 1.37E-03 | 5.43E-04 | 0.01 | 0.08 |
| R. Insula | 1.39E-03 | 5.62E-04 | 0.02 | 0.09 |
| L. Precentral | 1.31E-03 | 5.43E-04 | 0.02 | 0.09 |
| R. Pars Orbitalis | 1.67E-03 | 6.92E-04 | 0.02 | 0.09 |
| L. Superior Temporal | 1.24E-03 | 5.22E-04 | 0.02 | 0.09 |
| L. Precuneus | 1.11E-03 | 4.69E-04 | 0.02 | 0.09 |
| L. Medial Orbitofrontal | 1.17E-03 | 5.17E-04 | 0.03 | 0.10 |
| R. Pericalcarine | 1.23E-03 | 5.51E-04 | 0.03 | 0.10 |
| R. Inferior Temporal | 1.02E-03 | 4.59E-04 | 0.03 | 0.10 |
| R. Caudal Middle Frontal | 1.28E-03 | 5.82E-04 | 0.03 | 0.10 |
| R. Rostral Middle Frontal | 1.09E-03 | 4.96E-04 | 0.03 | 0.10 |
| L. Caudal Middle Frontal | 1.05E-03 | 5.12E-04 | 0.05 | 0.13 |
| R. Banks of the Superior Temporal Sulcus | 1.20E-03 | 5.91E-04 | 0.05 | 0.13 |
| L. Superior Frontal | 1.07E-03 | 5.30E-04 | 0.05 | 0.13 |
| L. Entorhinal | 2.13E-03 | 1.07E-03 | 0.05 | 0.13 |
| L. Postcentral | 9.24E-04 | 4.63E-04 | 0.05 | 0.13 |
| R. Paracentral | 1.05E-03 | 5.30E-04 | 0.05 | 0.13 |
| L. Lingual | 8.90E-04 | 4.57E-04 | 0.06 | 0.13 |
| R. Supramarginal | 1.01E-03 | 5.18E-04 | 0.06 | 0.13 |
| L. Banks of the Superior Temporal Sulcus | 8.90E-04 | 4.91E-04 | 0.07 | 0.17 |
| R. Temporal Pole | 2.31E-03 | 1.35E-03 | 0.09 | 0.20 |
| R. Precuneus | 7.68E-04 | 4.58E-04 | 0.10 | 0.20 |
| R. Middle Temporal | 8.69E-04 | 5.20E-04 | 0.10 | 0.20 |
| R. Postcentral | 8.22E-04 | 5.00E-04 | 0.10 | 0.21 |
| R. Lateral Orbitofrontal | 8.17E-04 | 5.01E-04 | 0.11 | 0.21 |
| R. Precentral | 1.02E-03 | 6.41E-04 | 0.12 | 0.22 |
| L. Temporal Pole | 2.07E-03 | 1.33E-03 | 0.12 | 0.23 |
| R. Pars Triangularis | 7.29E-04 | 4.76E-04 | 0.13 | 0.23 |
| R. Entorhinal | 1.93E-03 | 1.26E-03 | 0.13 | 0.23 |
| R. Cuneus | 7.18E-04 | 4.89E-04 | 0.15 | 0.25 |
| R. Inferior Parietal | 6.24E-04 | 4.57E-04 | 0.18 | 0.29 |
| L. Inferior Parietal | 4.90E-04 | 3.89E-04 | 0.21 | 0.34 |
| L. Posterior Cingulate | 7.16E-04 | 5.85E-04 | 0.23 | 0.36 |
| L. Lateral Occipital | 5.26E-04 | 4.45E-04 | 0.24 | 0.37 |
| R. Superior Parietal | 5.46E-04 | 4.72E-04 | 0.25 | 0.38 |
| L. Superior Parietal | 5.17E-04 | 4.50E-04 | 0.25 | 0.38 |
| L. Pericalcarine | 7.01E-04 | 6.26E-04 | 0.27 | 0.38 |
| L. Supramarginal | 5.52E-04 | 4.95E-04 | 0.27 | 0.38 |
| R. Fusiform | 4.14E-04 | 3.86E-04 | 0.29 | 0.39 |
| R. Transverse Temporal | 8.20E-04 | 7.69E-04 | 0.29 | 0.39 |
| R. Lateral Occipital | 4.33E-04 | 4.16E-04 | 0.30 | 0.40 |
| L. Cuneus | 5.99E-04 | 5.80E-04 | 0.31 | 0.40 |
| L. Fusiform | 3.72E-04 | 4.07E-04 | 0.36 | 0.47 |
| L. Middle Temporal | 4.05E-04 | 4.91E-04 | 0.41 | 0.52 |
| L. Parahippocampal | -5.41E-04 | 8.26E-04 | 0.51 | 0.64 |
| L. Rostral Anterior Cingulate | -3.29E-04 | 6.54E-04 | 0.62 | 0.75 |
| R. Isthmus Cingulate | -2.50E-04 | 5.64E-04 | 0.66 | 0.79 |
| L. Isthmus Cingulate | -2.61E-04 | 6.47E-04 | 0.69 | 0.79 |
| R. Posterior Cingulate | -1.87E-04 | 4.68E-04 | 0.69 | 0.79 |
| L. Inferior Temporal | 1.67E-04 | 4.28E-04 | 0.70 | 0.79 |
| R. Caudal Anterior Cingulate | -2.10E-04 | 7.08E-04 | 0.77 | 0.85 |
| R. Parahippocampal | -2.30E-04 | 8.16E-04 | 0.78 | 0.85 |
| L. Frontal Pole | 1.90E-04 | 8.29E-04 | 0.82 | 0.88 |
| R. Rostral Anterior Cingulate | -1.46E-04 | 7.38E-04 | 0.84 | 0.89 |
| L. Paracentral | -1.11E-04 | 5.99E-04 | 0.85 | 0.89 |
| R. Frontal Pole | 1.61E-04 | 9.96E-04 | 0.87 | 0.89 |
| L. Transverse Temporal | -1.20E-04 | 7.47E-04 | 0.87 | 0.89 |
| L. Caudal Anterior Cingulate | -3.15E-05 | 6.25E-04 | 0.96 | 0.96 |

Associations between right mediodorsal lateral parvocellular nucleus volumes and cortical thicknesses are shown. Results for all 68 cortical regions of interest from the Desikan-Killiany atlas with *p*-values shown before and after FDR correction for multiple testing. All regression analysis covaried for clinical severity (as estimated by CDR-SB score), age, sex, education, MRI scanner type (1.5T, 3T, or 4T), and total intracranial volume. R. – Right, L. – Left.
