# Supplementary material for: Radiogenomics of C9orf72 Expansion Carriers Reveals Global Transposable Element Derepression and Enables Prediction of Thalamic Atrophy and Clinical Impairment
Source: J Neurosci. 2023 Jan 11;43(2):333–45. doi: 10.1523/JNEUROSCI.1448-22.2022 (PMC9838702; doi:10.1523/JNEUROSCI.1448-22.2022)
Supplement: Figure 3-3 — CDR-SB score associations with whole-brain cortical thicknesses. Associations between CDR-SB score and cortical thicknesses are shown. Results for all 68 cortical regions of interest from the Desikan–Killiany atlas with associated p values shown before and after FDR correction for multiple testing. All regression analysis covaried for age, sex, education, MRI scanner type (1.5T, 3T, or 4T), and total intracranial volume. L, Left. Download Figure 3-3, DOCX file. [file ns-JN-RM-1448-22-s05.docx]

Figure 3-3: CDR-SB score associations with whole-brain cortical thicknesses

| Region | Beta | Standard Error | *P*-Value | FDR *P*-Value |
| --- | --- | --- | --- | --- |
| L. Pars Triangularis | -3.55E-02 | 4.73E-03 | 1.57E-10 | 1.07E-08 |
| R. Middle Temporal | -2.99E-02 | 4.61E-03 | 1.12E-08 | 3.81E-07 |
| L. Pars Opercularis | -3.08E-02 | 4.92E-03 | 2.89E-08 | 5.34E-07 |
| L. Middle Temporal | -2.67E-02 | 4.29E-03 | 3.14E-08 | 5.34E-07 |
| R. Pars Opercularis | -2.80E-02 | 4.58E-03 | 5.09E-08 | 6.92E-07 |
| R. Superior Frontal | -2.63E-02 | 4.38E-03 | 7.85E-08 | 8.90E-07 |
| R. Pars Triangularis | -2.48E-02 | 4.20E-03 | 1.18E-07 | 1.15E-06 |
| L. Lateral Orbitofrontal | -2.40E-02 | 4.14E-03 | 1.79E-07 | 1.52E-06 |
| R. Inferior Temporal | -2.31E-02 | 4.13E-03 | 4.10E-07 | 3.10E-06 |
| L. Caudal Middle Frontal | -2.53E-02 | 4.58E-03 | 5.74E-07 | 3.90E-06 |
| L. Superior Frontal | -2.59E-02 | 4.74E-03 | 6.94E-07 | 4.29E-06 |
| L. Precuneus | -2.30E-02 | 4.24E-03 | 8.41E-07 | 4.77E-06 |
| L. Inferior Parietal | -1.77E-02 | 3.42E-03 | 2.22E-06 | 1.16E-05 |
| L. Supramarginal | -2.21E-02 | 4.34E-03 | 2.81E-06 | 1.31E-05 |
| R. Caudal Middle Frontal | -2.67E-02 | 5.24E-03 | 2.88E-06 | 1.31E-05 |
| R. Fusiform | -1.61E-02 | 3.38E-03 | 1.06E-05 | 4.51E-05 |
| L. Rostral Middle Frontal | -2.21E-02 | 4.76E-03 | 1.56E-05 | 6.24E-05 |
| L. Inferior Temporal | -1.71E-02 | 3.73E-03 | 1.85E-05 | 6.99E-05 |
| L. Pars Orbitalis | -3.00E-02 | 6.55E-03 | 2.05E-05 | 7.34E-05 |
| L. Superior Parietal | -1.78E-02 | 3.95E-03 | 2.69E-05 | 9.15E-05 |
| L. Superior Temporal | -2.10E-02 | 4.72E-03 | 3.26E-05 | 1.06E-04 |
| R. Supramarginal | -1.97E-02 | 4.62E-03 | 6.33E-05 | 1.96E-04 |
| L. Banks of the Superior Temporal Sulcus | -1.85E-02 | 4.37E-03 | 6.72E-05 | 1.97E-04 |
| R. Superior Temporal | -1.83E-02 | 4.32E-03 | 6.97E-05 | 1.97E-04 |
| L. Medial Orbitofrontal | -1.96E-02 | 4.66E-03 | 7.60E-05 | 2.07E-04 |
| R. Precuneus | -1.67E-02 | 4.06E-03 | 1.05E-04 | 2.75E-04 |
| L. Entorhinal | -3.89E-02 | 9.55E-03 | 1.24E-04 | 3.12E-04 |
| R. Superior Parietal | -1.67E-02 | 4.14E-03 | 1.37E-04 | 3.34E-04 |
| R. Inferior Parietal | -1.62E-02 | 4.02E-03 | 1.45E-04 | 3.40E-04 |
| L. Precentral | -1.96E-02 | 4.92E-03 | 1.61E-04 | 3.64E-04 |
| R. Pars Orbitalis | -2.49E-02 | 6.27E-03 | 1.68E-04 | 3.69E-04 |
| R. Rostral Middle Frontal | -1.77E-02 | 4.46E-03 | 1.76E-04 | 3.73E-04 |
| R. Lateral Orbitofrontal | -1.73E-02 | 4.44E-03 | 2.28E-04 | 4.69E-04 |
| L. Postcentral | -1.60E-02 | 4.14E-03 | 2.48E-04 | 4.96E-04 |
| R. Posterior Cingulate | -1.54E-02 | 4.07E-03 | 3.23E-04 | 6.28E-04 |
| R. Postcentral | -1.57E-02 | 4.43E-03 | 7.05E-04 | 1.33E-03 |
| R. Precentral | -1.98E-02 | 5.67E-03 | 8.27E-04 | 1.52E-03 |
| R. Lateral Occipital | -1.27E-02 | 3.64E-03 | 8.74E-04 | 1.56E-03 |
| R. Insula | -1.64E-02 | 5.09E-03 | 1.91E-03 | 3.33E-03 |
| R. Banks of the Superior Temporal Sulcus | -1.66E-02 | 5.28E-03 | 2.49E-03 | 4.23E-03 |
| R. Entorhinal | -3.31E-02 | 1.11E-02 | 4.07E-03 | 6.61E-03 |
| R. Temporal Pole | -3.55E-02 | 1.20E-02 | 4.08E-03 | 6.61E-03 |
| L. Insula | -1.44E-02 | 5.22E-03 | 7.44E-03 | 0.01 |
| R. Medial Orbitofrontal | -1.32E-02 | 4.93E-03 | 9.56E-03 | 0.01 |
| L. Posterior Cingulate | -1.37E-02 | 5.14E-03 | 9.71E-03 | 0.01 |
| R. Frontal Pole | -2.21E-02 | 8.66E-03 | 0.01 | 0.02 |
| R. Isthmus Cingulate | -1.26E-02 | 4.91E-03 | 0.01 | 0.02 |
| L. Lateral Occipital | -9.90E-03 | 3.91E-03 | 0.01 | 0.02 |
| L. Fusiform | -8.19E-03 | 3.55E-03 | 0.02 | 0.03 |
| L. Frontal Pole | -1.65E-02 | 7.20E-03 | 0.03 | 0.03 |
| R. Paracentral | -1.08E-02 | 4.74E-03 | 0.03 | 0.03 |
| L. Isthmus Cingulate | -1.25E-02 | 5.63E-03 | 0.03 | 0.04 |
| L. Paracentral | -9.84E-03 | 5.21E-03 | 0.06 | 0.08 |
| L. Temporal Pole | -2.02E-02 | 1.18E-02 | 0.09 | 0.11 |
| L. Cuneus | -8.27E-03 | 5.08E-03 | 0.11 | 0.13 |
| L. Lingual | -6.06E-03 | 4.08E-03 | 0.14 | 0.17 |
| R. Lingual | -5.50E-03 | 3.78E-03 | 0.15 | 0.18 |
| L. Pericalcarine | -6.59E-03 | 5.49E-03 | 0.23 | 0.27 |
| R. Cuneus | -5.07E-03 | 4.31E-03 | 0.24 | 0.28 |
| L. Parahippocampal | -7.29E-03 | 7.20E-03 | 0.31 | 0.36 |
| R. Rostral Anterior Cingulate | -5.73E-03 | 6.41E-03 | 0.37 | 0.42 |
| L. Rostral Anterior Cingulate | -4.21E-03 | 5.69E-03 | 0.46 | 0.51 |
| L. Caudal Anterior Cingulate | -3.69E-03 | 5.43E-03 | 0.50 | 0.54 |
| R. Parahippocampal | -4.69E-03 | 7.09E-03 | 0.51 | 0.54 |
| R. Transverse Temporal | -3.92E-03 | 6.73E-03 | 0.56 | 0.59 |
| R. Caudal Anterior Cingulate | -2.71E-03 | 6.15E-03 | 0.66 | 0.68 |
| L. Transverse Temporal | -1.68E-03 | 6.49E-03 | 0.80 | 0.81 |
| R. Pericalcarine | -8.15E-04 | 4.96E-03 | 0.87 | 0.87 |

Associations between CDR-SB score and cortical thicknesses are shown. Results for all 68 cortical regions of interest from the Desikan-Killiany atlas with associated *p*-values shown before and after FDR correction for multiple testing. All regression analysis covaried for age, sex, education, MRI scanner type (1.5T, 3T, or 4T), and total intracranial volume. R. – Right, L. – Left.
