# Supplementary material for: Radiogenomics of C9orf72 Expansion Carriers Reveals Global Transposable Element Derepression and Enables Prediction of Thalamic Atrophy and Clinical Impairment
Source: J Neurosci. 2023 Jan 11;43(2):333–45. doi: 10.1523/JNEUROSCI.1448-22.2022 (PMC9838702; doi:10.1523/JNEUROSCI.1448-22.2022)
Supplement: Figure 3-4 — Cortical thickness differences in C9orf72 HRE carriers compared to controls. Associations between C9orf72 HRE carrier status and cortical thicknesses are shown for all 68 cortical regions of interest from the Desikan–Killiany atlas with associated p values shown before and after FDR correction for multiple testing. All regression analysis covaried for clinical severity (as estimated by CDR-SB score), age, sex, education, MRI scanner type (1.5T, 3T, or 4T), and total intracranial volume. L, Left. Download Figure 3-4, DOCX file. [file ns-JN-RM-1448-22-s06.docx]

Figure 3-4: Cortical thickness differences in *C9orf72* HRE carriers compared to controls

| Region | Beta | Standard Error | *P*-Value | FDR *P*-Value |
| --- | --- | --- | --- | --- |
| R. Pericalcarine | -0.18 | 0.05 | 2.45E-04 | 0.01 |
| L. Rostral Middle Frontal | -0.17 | 0.04 | 4.27E-04 | 0.01 |
| R. Medial Orbitofrontal | -0.15 | 0.05 | 1.76E-03 | 0.03 |
| R. Superior Frontal | -0.14 | 0.04 | 1.97E-03 | 0.03 |
| L. Inferior Parietal | -0.11 | 0.03 | 2.16E-03 | 0.03 |
| R. Caudal Middle Frontal | -0.16 | 0.05 | 2.94E-03 | 0.03 |
| L. Caudal Middle Frontal | -0.14 | 0.04 | 3.15E-03 | 0.03 |
| L. Superior Parietal | -0.12 | 0.04 | 3.62E-03 | 0.03 |
| L. Pericalcarine | -0.16 | 0.05 | 4.61E-03 | 0.03 |
| L. Lateral Occipital | -0.11 | 0.04 | 6.71E-03 | 0.04 |
| R. Postcentral | -0.12 | 0.04 | 7.17E-03 | 0.04 |
| R. Rostral Middle Frontal | -0.12 | 0.04 | 7.25E-03 | 0.04 |
| L. Postcentral | -0.11 | 0.04 | 7.60E-03 | 0.04 |
| L. Superior Frontal | -0.13 | 0.05 | 8.56E-03 | 0.04 |
| L. Precuneus | -0.11 | 0.04 | 8.80E-03 | 0.04 |
| L. Pars Opercularis | -0.13 | 0.05 | 8.97E-03 | 0.04 |
| L. Supramarginal | -0.11 | 0.04 | 0.01 | 0.04 |
| R. Superior Temporal | -0.11 | 0.04 | 0.01 | 0.04 |
| R. Superior Parietal | -0.10 | 0.04 | 0.01 | 0.05 |
| R. Lateral Occipital | -0.09 | 0.04 | 0.01 | 0.05 |
| R. Precuneus | -0.10 | 0.04 | 0.02 | 0.06 |
| R. Inferior Parietal | -0.09 | 0.04 | 0.02 | 0.07 |
| R. Lingual | -0.09 | 0.04 | 0.02 | 0.07 |
| R. Pars Opercularis | -0.10 | 0.05 | 0.02 | 0.07 |
| L. Lingual | -0.09 | 0.04 | 0.03 | 0.08 |
| R. Supramarginal | -0.10 | 0.05 | 0.03 | 0.08 |
| L. Medial Orbitofrontal | -0.10 | 0.05 | 0.03 | 0.08 |
| R. Cuneus | -0.09 | 0.04 | 0.03 | 0.08 |
| L. Banks of the Superior Temporal Sulcus | -0.09 | 0.04 | 0.04 | 0.09 |
| R. Paracentral | -0.10 | 0.05 | 0.05 | 0.11 |
| L. Posterior Cingulate | -0.10 | 0.05 | 0.05 | 0.11 |
| L. Lateral Orbitofrontal | -0.08 | 0.04 | 0.05 | 0.11 |
| L. Superior Temporal | -0.09 | 0.05 | 0.06 | 0.11 |
| L. Precentral | -0.10 | 0.05 | 0.06 | 0.11 |
| R. Inferior Temporal | -0.08 | 0.04 | 0.06 | 0.11 |
| L. Pars Triangularis | -0.09 | 0.05 | 0.08 | 0.15 |
| L. Pars Orbitalis | -0.11 | 0.07 | 0.11 | 0.20 |
| R. Middle Temporal | -0.07 | 0.05 | 0.12 | 0.20 |
| R. Transverse Temporal | -0.11 | 0.07 | 0.12 | 0.20 |
| R. Lateral Orbitofrontal | -0.07 | 0.05 | 0.12 | 0.20 |
| L. Insula | -0.08 | 0.05 | 0.13 | 0.21 |
| R. Pars Orbitalis | -0.10 | 0.06 | 0.13 | 0.21 |
| R. Temporal Pole | -0.18 | 0.12 | 0.14 | 0.22 |
| L. Entorhinal | -0.14 | 0.10 | 0.14 | 0.22 |
| L. Temporal Pole | -0.16 | 0.12 | 0.18 | 0.27 |
| L. Middle Temporal | -0.06 | 0.04 | 0.19 | 0.28 |
| R. Frontal Pole | -0.11 | 0.09 | 0.20 | 0.29 |
| R. Banks of the Superior Temporal Sulcus | -0.07 | 0.05 | 0.21 | 0.29 |
| R. Pars Triangularis | -0.05 | 0.04 | 0.21 | 0.29 |
| R. Precentral | -0.07 | 0.06 | 0.22 | 0.30 |
| R. Insula | -0.06 | 0.05 | 0.24 | 0.32 |
| L. Paracentral | -0.06 | 0.05 | 0.26 | 0.34 |
| L. Cuneus | -0.06 | 0.05 | 0.28 | 0.35 |
| L. Rostral Anterior Cingulate | -0.06 | 0.06 | 0.29 | 0.36 |
| R. Posterior Cingulate | -0.04 | 0.04 | 0.31 | 0.38 |
| L. Fusiform | -0.04 | 0.04 | 0.34 | 0.41 |
| R. Fusiform | -0.03 | 0.03 | 0.41 | 0.49 |
| L. Frontal Pole | -0.05 | 0.07 | 0.48 | 0.56 |
| R. Caudal Anterior Cingulate | 0.03 | 0.06 | 0.63 | 0.72 |
| L. Transverse Temporal | -0.03 | 0.07 | 0.66 | 0.74 |
| L. Parahippocampal | 0.02 | 0.07 | 0.82 | 0.90 |
| R. Entorhinal | -0.03 | 0.11 | 0.83 | 0.90 |
| R. Parahippocampal | -0.02 | 0.07 | 0.83 | 0.90 |
| L. Caudal Anterior Cingulate | 9.55E-03 | 0.06 | 0.87 | 0.92 |
| R. Isthmus Cingulate | -5.72E-03 | 0.05 | 0.91 | 0.95 |
| R. Rostral Anterior Cingulate | -6.01E-03 | 0.07 | 0.93 | 0.95 |
| L. Inferior Temporal | 2.97E-03 | 0.04 | 0.94 | 0.95 |
| L. Isthmus Cingulate | -3.76E-04 | 0.06 | 0.99 | 0.99 |

Associations between *C9orf72* HRE carrier status and cortical thicknesses are shown for all 68 cortical regions of interest from the Desikan-Killiany atlas with associated *p*-values shown before and after FDR correction for multiple testing. All regression analysis covaried for clinical severity (as estimated by CDR-SB score), age, sex, education, MRI scanner type (1.5T, 3T, or 4T), and total intracranial volume. R. – Right, L. – Left.
