# Supplementary material for: Radiogenomics of C9orf72 Expansion Carriers Reveals Global Transposable Element Derepression and Enables Prediction of Thalamic Atrophy and Clinical Impairment
Source: J Neurosci. 2023 Jan 11;43(2):333–45. doi: 10.1523/JNEUROSCI.1448-22.2022 (PMC9838702; doi:10.1523/JNEUROSCI.1448-22.2022)
Supplement: Figure 3-5 — Cortical thickness associations with C9orf72 expression in HRE carriers and controls. Associations of C9orf72 expression and cortical thicknesses are shown for all 68 cortical regions of interest from the Desikan–Killiany atlas with associated p values shown before and after FDR correction for multiple testing. All regression analysis covaried for clinical severity (as estimated by CDR-SB score), age, sex, education, MRI scanner type (1.5T, 3T, or 4T), and total intracranial volume. L, Left. Download Figure 3-5, DOCX file. [file ns-JN-RM-1448-22-s07.docx]

Figure 3-5: Cortical thickness associations with *C9orf72* expression in HRE carriers and controls

| Region | Beta | Standard Error | *P*-Value | FDR *P*-Value |
| --- | --- | --- | --- | --- |
| L. Pars Triangularis | 0.10 | 0.03 | 2.54E-03 | 0.08 |
| L. Precuneus | 0.09 | 0.03 | 3.29E-03 | 0.08 |
| L. Rostral Middle Frontal | 0.09 | 0.03 | 3.61E-03 | 0.08 |
| L. Lateral Orbitofrontal | 0.08 | 0.03 | 4.48E-03 | 0.08 |
| L. Medial Orbitofrontal | 0.09 | 0.03 | 5.68E-03 | 0.08 |
| L. Superior Parietal | 0.07 | 0.03 | 0.01 | 0.10 |
| L. Pars Opercularis | 0.09 | 0.03 | 0.01 | 0.10 |
| L. Superior Frontal | 0.08 | 0.03 | 0.01 | 0.10 |
| L. Caudal Middle Frontal | 0.08 | 0.03 | 0.02 | 0.11 |
| R. Fusiform | 0.06 | 0.02 | 0.02 | 0.11 |
| R. Pars Opercularis | 0.07 | 0.03 | 0.03 | 0.15 |
| L. Postcentral | 0.06 | 0.03 | 0.03 | 0.15 |
| L. Pars Orbitalis | 0.09 | 0.05 | 0.06 | 0.26 |
| L. Frontal Pole | 0.10 | 0.05 | 0.06 | 0.26 |
| R. Rostral Middle Frontal | 0.06 | 0.03 | 0.06 | 0.26 |
| R. Superior Temporal | 0.06 | 0.03 | 0.06 | 0.26 |
| R. Middle Temporal | 0.06 | 0.03 | 0.07 | 0.26 |
| R. Pars Triangularis | 0.05 | 0.03 | 0.07 | 0.26 |
| R. Superior Parietal | 0.05 | 0.03 | 0.07 | 0.26 |
| L. Lateral Occipital | 0.05 | 0.03 | 0.08 | 0.29 |
| L. Pericalcarine | 0.06 | 0.04 | 0.09 | 0.29 |
| R. Superior Frontal | 0.05 | 0.03 | 0.10 | 0.29 |
| L. Supramarginal | 0.05 | 0.03 | 0.10 | 0.29 |
| R. Medial Orbitofrontal | 0.06 | 0.03 | 0.10 | 0.29 |
| R. Postcentral | 0.05 | 0.03 | 0.12 | 0.30 |
| R. Rostral Anterior Cingulate | -0.07 | 0.04 | 0.12 | 0.30 |
| L. Middle Temporal | 0.05 | 0.03 | 0.12 | 0.30 |
| R. Lingual | 0.04 | 0.03 | 0.13 | 0.32 |
| R. Inferior Parietal | 0.04 | 0.03 | 0.14 | 0.32 |
| R. Posterior Cingulate | 0.04 | 0.03 | 0.15 | 0.33 |
| L. Inferior Parietal | 0.03 | 0.02 | 0.17 | 0.37 |
| R. Caudal Middle Frontal | 0.05 | 0.04 | 0.18 | 0.37 |
| L. Superior Temporal | 0.04 | 0.03 | 0.19 | 0.37 |
| L. Precentral | 0.05 | 0.03 | 0.19 | 0.37 |
| L. Posterior Cingulate | 0.05 | 0.04 | 0.19 | 0.37 |
| L. Cuneus | 0.04 | 0.04 | 0.22 | 0.42 |
| R. Precuneus | 0.03 | 0.03 | 0.23 | 0.42 |
| R. Temporal Pole | 0.10 | 0.08 | 0.24 | 0.44 |
| L. Rostral Anterior Cingulate | 0.05 | 0.04 | 0.25 | 0.44 |
| R. Lateral Orbitofrontal | 0.04 | 0.03 | 0.26 | 0.44 |
| R. Banks of the Superior Temporal Sulcus | 0.04 | 0.04 | 0.27 | 0.44 |
| R. Entorhinal | 0.09 | 0.08 | 0.27 | 0.44 |
| L. Temporal Pole | 0.09 | 0.08 | 0.28 | 0.44 |
| L. Parahippocampal | -0.05 | 0.05 | 0.29 | 0.44 |
| R. Pericalcarine | 0.04 | 0.03 | 0.31 | 0.46 |
| L. Isthmus Cingulate | 0.04 | 0.04 | 0.31 | 0.46 |
| R. Lateral Occipital | 0.03 | 0.03 | 0.33 | 0.46 |
| R. Isthmus Cingulate | 0.03 | 0.03 | 0.33 | 0.46 |
| L. Banks of the Superior Temporal Sulcus | 0.03 | 0.03 | 0.37 | 0.51 |
| L. Fusiform | 0.02 | 0.03 | 0.42 | 0.55 |
| L. Inferior Temporal | 0.02 | 0.03 | 0.42 | 0.55 |
| L. Lingual | 0.02 | 0.03 | 0.43 | 0.55 |
| L. Insula | 0.03 | 0.04 | 0.43 | 0.55 |
| R. Pars Orbitalis | 0.03 | 0.04 | 0.44 | 0.55 |
| R. Paracentral | 0.02 | 0.03 | 0.49 | 0.61 |
| R. Inferior Temporal | 0.02 | 0.03 | 0.51 | 0.61 |
| L. Entorhinal | 0.04 | 0.07 | 0.53 | 0.64 |
| R. Supramarginal | 0.02 | 0.03 | 0.58 | 0.67 |
| L. Transverse Temporal | -0.03 | 0.05 | 0.59 | 0.67 |
| R. Insula | 0.02 | 0.04 | 0.59 | 0.67 |
| R. Caudal Anterior Cingulate | 0.02 | 0.04 | 0.63 | 0.71 |
| R. Parahippocampal | -0.02 | 0.05 | 0.73 | 0.80 |
| L. Paracentral | 0.01 | 0.04 | 0.78 | 0.84 |
| R. Transverse Temporal | 0.01 | 0.05 | 0.81 | 0.86 |
| R. Cuneus | 0.01 | 0.03 | 0.83 | 0.87 |
| L. Caudal Anterior Cingulate | 0.01 | 0.04 | 0.88 | 0.91 |
| R. Precentral | 4.39E-03 | 0.04 | 0.91 | 0.93 |
| R. Frontal Pole | -3.69E-03 | 0.06 | 0.95 | 0.95 |

Associations of *C9orf72* expression and cortical thicknesses are shown for all 68 cortical regions of interest from the Desikan-Killiany atlas with associated *p*-values shown before and after FDR correction for multiple testing. All regression analysis covaried for clinical severity (as estimated by CDR-SB score), age, sex, education, MRI scanner type (1.5T, 3T, or 4T), and total intracranial volume. R. – Right, L. – Left.
