# Supplementary material for: Radiogenomics of C9orf72 Expansion Carriers Reveals Global Transposable Element Derepression and Enables Prediction of Thalamic Atrophy and Clinical Impairment
Source: J Neurosci. 2023 Jan 11;43(2):333–45. doi: 10.1523/JNEUROSCI.1448-22.2022 (PMC9838702; doi:10.1523/JNEUROSCI.1448-22.2022)
Supplement: Figure 4-3 — Thalamic volume associations with L1HS expression. Associations of thalamic nuclei volumes with L1HS expression in a combined cohort of C9orf72 HRE carriers and controls. Results from all 50 thalamic nuclei volumes estimated using FreeSurfer 7.1 software are shown above with p values shown before and after FDR correction for multiple testing. All regression analysis covaried for clinical severity (as estimated by CDR-SB score), age, sex, education, MRI scanner type (1.5T, 3T, or 4T), and total intracranial volume. L, Left. Download Figure 4-3, DOCX file. [file ns-JN-RM-1448-22-s10.docx]

Figure 4-3: Thalamic volume associations with *L1HS* expression

| Region | Beta | SE | *P*-Val | FDR *P*-val |
| --- | --- | --- | --- | --- |
| L. Pulvinar Lateral | -8.20 | 2.50 | 1.64E-03 | 0.08 |
| R. Pulvinar Medial | -30.30 | 10.95 | 7.31E-03 | 0.13 |
| R. Pulvinar Lateral | -7.08 | 2.58 | 7.86E-03 | 0.13 |
| R. Pulvinar Anterior | -5.60 | 2.18 | 0.01 | 0.16 |
| L. Pulvinar Medial | -21.77 | 9.51 | 0.03 | 0.25 |
| R. Lateral Posterior | -4.35 | 1.97 | 0.03 | 0.25 |
| L. Suprageniculate | -1.19 | 0.57 | 0.04 | 0.28 |
| L. Pulvinar Anterior | -4.02 | 2.00 | 0.05 | 0.30 |
| R. Ventromedial | -0.63 | 0.37 | 0.10 | 0.50 |
| L. Lateral Posterior | -3.38 | 2.04 | 0.10 | 0.50 |
| L. Ventromedial | -0.52 | 0.32 | 0.11 | 0.50 |
| L. Medial Geniculate | -2.23 | 1.45 | 0.13 | 0.52 |
| R. Pulvinar Inferior | -4.61 | 3.06 | 0.14 | 0.52 |
| R. Intralaminar Central Lateral | -1.10 | 0.77 | 0.16 | 0.55 |
| R. Medial Geniculate | -2.63 | 1.88 | 0.17 | 0.55 |
| L. Pulvinar Inferior | -3.27 | 2.45 | 0.19 | 0.57 |
| R. Suprageniculate | -0.68 | 0.52 | 0.19 | 0.57 |
| L. Ventral Lateral Anterior | -6.18 | 4.92 | 0.21 | 0.58 |
| R. Laterodorsal | -1.11 | 0.92 | 0.23 | 0.58 |
| R. Mediodorsal Lateral Parvocellular | -3.60 | 2.99 | 0.23 | 0.58 |
| L. Ventral Anterior | -4.44 | 3.76 | 0.24 | 0.58 |
| L. Ventral Anterior Magnocellular | -0.34 | 0.30 | 0.25 | 0.58 |
| L. Lateral Geniculate | -2.85 | 2.58 | 0.27 | 0.59 |
| R. Anteroventral | -2.37 | 2.20 | 0.28 | 0.59 |
| R. Ventral Anterior | -3.80 | 3.76 | 0.32 | 0.63 |
| L. Mediodorsal Lateral Parvocellular | -2.83 | 2.91 | 0.33 | 0.63 |
| R. Intralaminar Central Medial | -0.90 | 0.94 | 0.34 | 0.63 |
| L. Ventral Lateral Posterior | -5.44 | 6.12 | 0.38 | 0.63 |
| L. Parafascicular | 0.48 | 0.54 | 0.38 | 0.63 |
| L. Mediodorsal Medial Magnocellular | 6.92 | 7.91 | 0.38 | 0.63 |
| R. Lateral Geniculate | -2.25 | 2.60 | 0.39 | 0.63 |
| L. Laterodorsal | -0.67 | 0.83 | 0.42 | 0.66 |
| L. Medial Ventral (Reuniens) | -0.18 | 0.23 | 0.44 | 0.67 |
| L. Paratenial | 0.06 | 0.08 | 0.47 | 0.68 |
| R. Medial Ventral (Reuniens) | -0.19 | 0.27 | 0.48 | 0.68 |
| R. Ventral Posterolateral | -5.88 | 8.79 | 0.51 | 0.68 |
| L. Anteroventral | -1.48 | 2.24 | 0.51 | 0.68 |
| R. Ventral Anterior Magnocellular | -0.20 | 0.31 | 0.52 | 0.68 |
| R. Ventral Lateral Anterior | -2.86 | 4.81 | 0.55 | 0.71 |
| L. Ventral Posterolateral | -4.52 | 7.90 | 0.57 | 0.71 |
| R. Intralaminar Centromedian | -1.18 | 2.28 | 0.61 | 0.74 |
| L. Paracentral | -0.02 | 0.04 | 0.63 | 0.75 |
| R. Paracentral | 0.02 | 0.04 | 0.73 | 0.82 |
| R. Ventral Lateral Posterior | -2.07 | 5.91 | 0.73 | 0.82 |
| L. Intralaminar Central Medial | -0.32 | 0.96 | 0.74 | 0.82 |
| L. Intralaminar Centromedian | 0.68 | 2.21 | 0.76 | 0.83 |
| L. Intralaminar Central Lateral | -0.07 | 0.69 | 0.92 | 0.97 |
| R. Parafascicular | 0.05 | 0.63 | 0.94 | 0.97 |
| R. Mediodorsal Medial Magnocellular | -0.35 | 7.09 | 0.96 | 0.97 |
| R. Paratenial | 0.00 | 0.08 | 0.97 | 0.97 |

Associations of thalamic nuclei volumes with *L1HS* expression in a combined cohort of *C9orf72* HRE carriers and controls. Results from all 50 thalamic nuclei volumes estimated using Freesurfer 7.1 are shown above with *p*-values shown before and after FDR correction for multiple testing. All regression analysis covaried for clinical severity (as estimated by CDR-SB score), age, sex, education, MRI scanner type (1.5T, 3T, or 4T), and total intracranial volume. R. – Right, L. – Left.
