# Supplementary material for: Radiogenomics of C9orf72 Expansion Carriers Reveals Global Transposable Element Derepression and Enables Prediction of Thalamic Atrophy and Clinical Impairment
Source: J Neurosci. 2023 Jan 11;43(2):333–45. doi: 10.1523/JNEUROSCI.1448-22.2022 (PMC9838702; doi:10.1523/JNEUROSCI.1448-22.2022)
Supplement: Figure 5-1 — Demographic characteristics of PBMC RNA-seq cohort. Download Figure 5-1, DOCX file. [file ns-JN-RM-1448-22-s11.docx]

**Figure 5-1.** Demographic characteristics of PBMC RNA-seq cohort.

|  | Control | *C9orf72* HRE ALS | Sporadic ALS |
| --- | --- | --- | --- |
| *n* | 8 | 10 | 10 |
| Age, years (mean [SD]) | 51.5 (7.0) | 57.7 (8.4) | 51.3 (5.8) |
| Sex, *n* male (%) | 5 (62.5) | 4 (40.0) | 5 (50.0) |

ALS, amyotrophic lateral sclerosis. HRE, hexanucleotide repeat expansion.
